# A Bibliometric and Visual Analysis of Nanocomposite Hydrogels Based on VOSviewer From 2010 to 2022

**DOI:** 10.3389/fbioe.2022.914253

**Published:** 2022-06-22

**Authors:** Mingyi Zhao, Hanqi Zhang, Zixin Li

**Affiliations:** ^1^ Department of Pediatrics, The Third Xiangya Hospital, Central South University, Changsha, China; ^2^ Xiangya School of Medicine, Central South University, Changsha, China

**Keywords:** bibliometrics, bibliometric analysis, nanocomposite hydrogels, biomedical application, publications

## Abstract

**Background:** Nanocomposite hydrogels (NHs) are stable composite materials formed by dispersing nanomaterials in hydrogels and have broad development prospects in the biomedical field. In this study, we aimed to systematically and comprehensively evaluate the trends and hot spots of biomedical applications of NHs from 2010 to 2022.

**Methods:** In total, 713 articles and reviews related to NH applications in the biomedical field from 2010 to 2022 were retrieved from the Web of Science Core Collection (WOSCC). Two scientometric software programs, VOSviewer and Microsoft Excel 2019, were used to visually perform bibliometric analysis in terms of research trends, sources, the contribution of journals, co-citation, and the co-occurrence of keywords.

**Results:** From 1 January 2010 to 3 February 2022, the number of annual scientific publications about NHs exhibited an upward trend, and research articles were published in a larger proportion (more than 77%). The top three countries in NH research were China, the United States, and India. Meanwhile, Tabriz University of Medical Sciences, the Chinese Academy of Sciences, and Tshwane University of Technology were the most active and contributive. In the contribution of journals, the journal *Advanced Functional Materials* had the highest number of publications, and the journal *Int J Biol Macro* had the most citations. Varaprasad K was the most prolific author, and Haraguchi K ranked first among co-cited authors. In the ranking of frequency in the co-cited references, Nanocomposite Hydrogels for Biomedical Applications, published by Gaharwar AK, was the most frequently cited reference. The keyword with the highest frequency was “drug delivery.”

**Conclusion:** This study performed a full overview of NHs using bibliometrics and identified current trends and hot spots. This information may help researchers focusing on NHs to identify developments in this field.

## Introduction

NHs are 3D cross-linked hydrated polymeric networks formed by dispersing nanomaterials in a hydrogel, which were first reported in 2002 (Gaharwar et al., 2014; Zhao et al., 2020). They retain the softness and good biocompatibility brought by the 3D network fiber structure and improve directed structure formation at multiple scales, having superior mechanical and functional properties (*e.g.*, physical, electrical, chemical, and biological properties) (Zhao et al., 2020; Zhang Y. et al., 2021). Due to the excellent performance of NHs, this has been a research hot spot in the various fields of materials, biology, and medicine. For instance, the introduction of nanomaterials in NHs improves the material area ratio, providing more adhesion sites for protein (Motealleh and Kehr, 2017; Mellati et al., 2021). Therefore, it is recognized by some researchers as a highly efficient choice for drug carriers, whether via *in vivo* or *in vitro* delivery routes (Kehr et al., 2015; Du et al., 2021). In addition, NHs also have outstanding biomimetic properties and histocompatibility, so they can place some special tissues for clinical treatment, such as cartilage, skeletal muscle, and cardiac tissues (Asadi et al., 2018; Piantanida et al., 2019; Zhao et al., 2020; Peper et al., 2021; Wang et al., 2021).

Over the last decade, there have been continuous breakthroughs in NH applications, especially in the biomedical field. Consequently, an analysis of progress in this area would be helpful. A bibliometric analysis, a comprehensive statistical method based on publications, is necessary for organizing a large amount of information (Zhang J. et al., 2021). To further understand the development of NHs in the biomedical field, we comprehensively searched the relevant 713 papers from the Web of Science (WOS) Core Collection. Then, these publications were visually bibliometrically analyzed based on VOSviewer and Microsoft Excel 2019 in terms of research trends, sources, the contribution of journals, authors, co-citations, and the co-occurrence of keywords.

By collecting and summarizing current research, this effective and intuitional analysis found trends and hot spots in NH research, which can guide more in-depth and comprehensive studies related to NHs for scholars in this field.

## Materials and Methods

### Data Source and Search

The data used in this study are all from WOSCC, including the Science Citation Index Expanded (SCI-Expanded) and Social Sciences Citation Index (SSCI), and are limited from 1 January 2010 to 3 February 2022, since we found that the publications in this field before 2010 were so rare that they could not show representativeness. However, the publications between 1 January 2010 and 3 February 2022 reveal the rapid development of this field. This profile, although incomplete, produces a considerable amount of literature for further study. The following is the retrieval strategy: “(TS = nanocomposite hydrogel) AND ((TS = biological application) OR (TS = medical application) OR (TS = biomedical application)).” Then, applying filters, the literature type is limited to “ARTICLE” or “REVIEW,” and the language of publications is English. We finally obtained 714 publications (580 articles and 134 reviews) and imported the records into VOSviewer for analysis. The inclusion criteria used in this study are as follows:

1) The study is about NHs applied in the biomedical field.
2) The publication period is from 1 January 2010 to 3 February 2022.
3) The language is English.
4) The literature type is “ARTICLE” or “REVIEW.”

The exclusion criteria applied in this study are as follows:

1) The types of literature are conference, report, and letters.
2) The studies did not use NHs as the main body.
3) The literature did not show the application of NHs in biomedicine.
4) The language is not English.

The reasons why we use the methods mentioned previously are due to the WOS as a famous database that collects authoritative literature all over the world, especially in the case of medicine and the natural sciences; therefore, we think that the literature in WOS is representative. English is a universal language, so we consider that the literature in English is more standard and meaningful than the literature in other languages.

### Data Analysis Methods

VOSviewer (Leiden University, Leiden, Netherlands) is visualized software based on the Java platform, which is an available tool for creating accessible maps using bibliographic data (van Eck and Waltman, 2010). Compared with other visualized tools, it is much effective and powerful in data processing and map generation. It enables researchers to quickly understand the hot spots and developments in their research field of interest. We downloaded the files in plain-text form from the WOS database and imported them into VOSviewer and Microsoft Excel 2019 for evaluation and analysis. In this study, we analyzed research trends, sources, contribution of journals, co-citations, and co-occurrence of keywords.

## Results

### Trend and Annual Count

**FIGURE 1:**
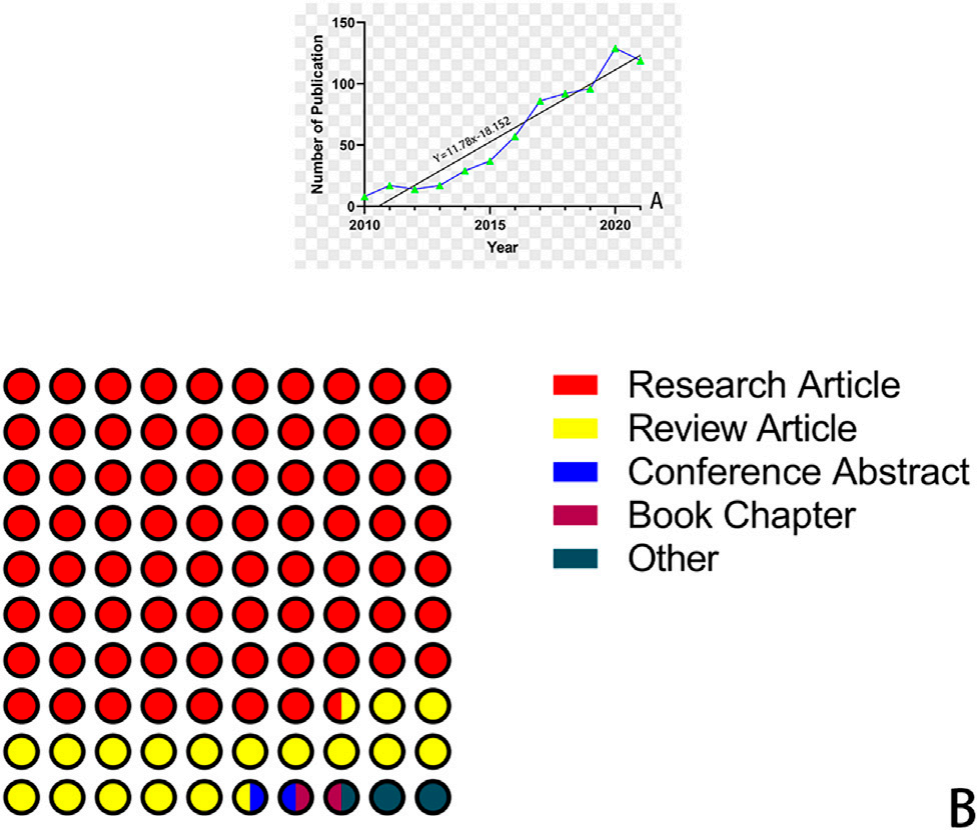
**(A)** Number of studies published in this field each year. **(B)** A number of studies of various types.

We counted the data of the WOS database from 2010 to 2022, with a total of 713 papers (including 578 research articles, 134 review articles, and 33 other types), as Figure 1A shows. Figure 1B indicates that the number of publications about nanocomposite hydrogels from 2010 to 2021, from 8 papers in 2010 to 119 papers in 2021, tends to increase year by year, with just a slight decrease in 2021. At the same time, by fitting the curve of the number of posts, the number of posts in this field approaches a linear relationship, which indicates that this field is in its initial stage and has great potential in the future. According to the information in the WOS database, the 713 papers were cited 22,721 times, for an average of 31.87 times per paper. We concluded that researchers are paying high attention to the application of nanocomposite hydrogels in biomedicine.

### Publication of Countries and Institutions

**FIGURE 2:**
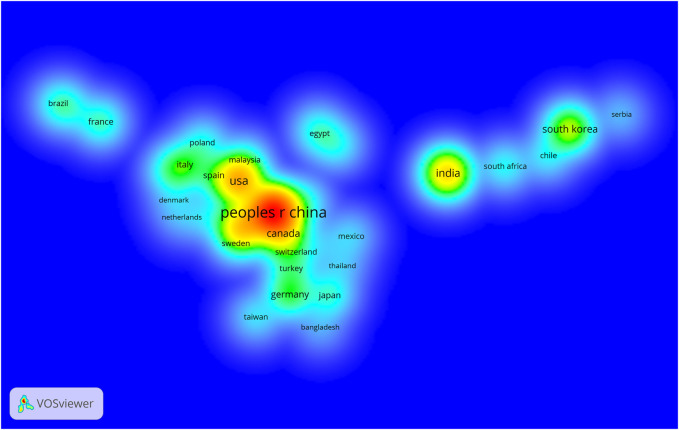
Visualized map of quantity of publication which is based on nanocomposite hydrogel and related research issued by countries and the density of co-author relationship.

**FIGURE 3:**
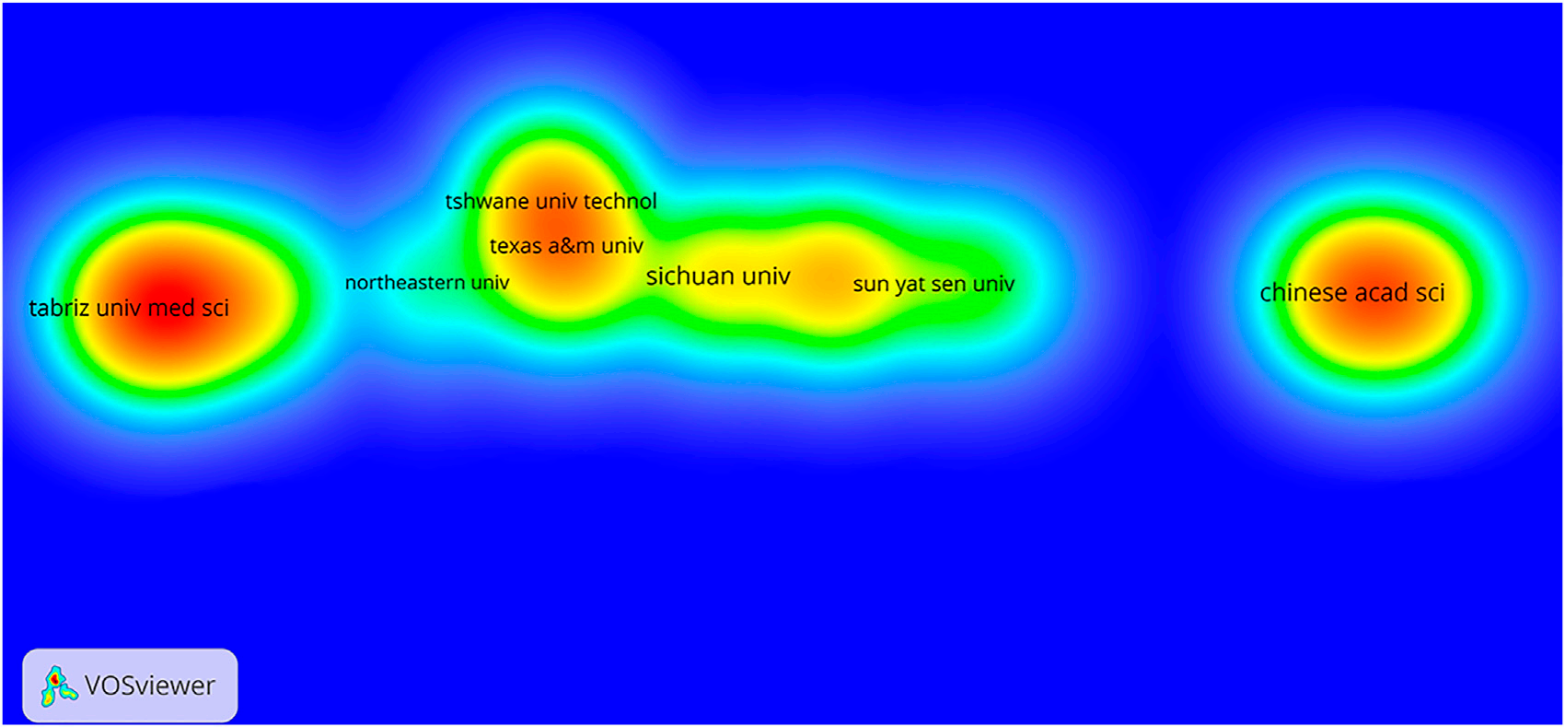
Visualized map of quantity of publication based on nanocomposite hydrogel and related research issued by organizations and the density of co-author relationship.

Figure 2 and Figure 3 show that many institutions and countries contributed to the number of publications on the application of nanocomposite hydrogels in biology and biomedicine from 2010 to 2022. Among them, the countries that made outstanding contributions were mainly China, the United States, and India, but there was not much cooperation among these countries. The institutions that made major contributions were *Tabriz University of Medical Sciences*, the *Chinese Academy of Sciences*, and *Tshwane University of Technology*. Meanwhile, the cooperation among these institutions in this field was relatively unaffiliated.

### The Contribution of Journals

**FIGURE 4:**
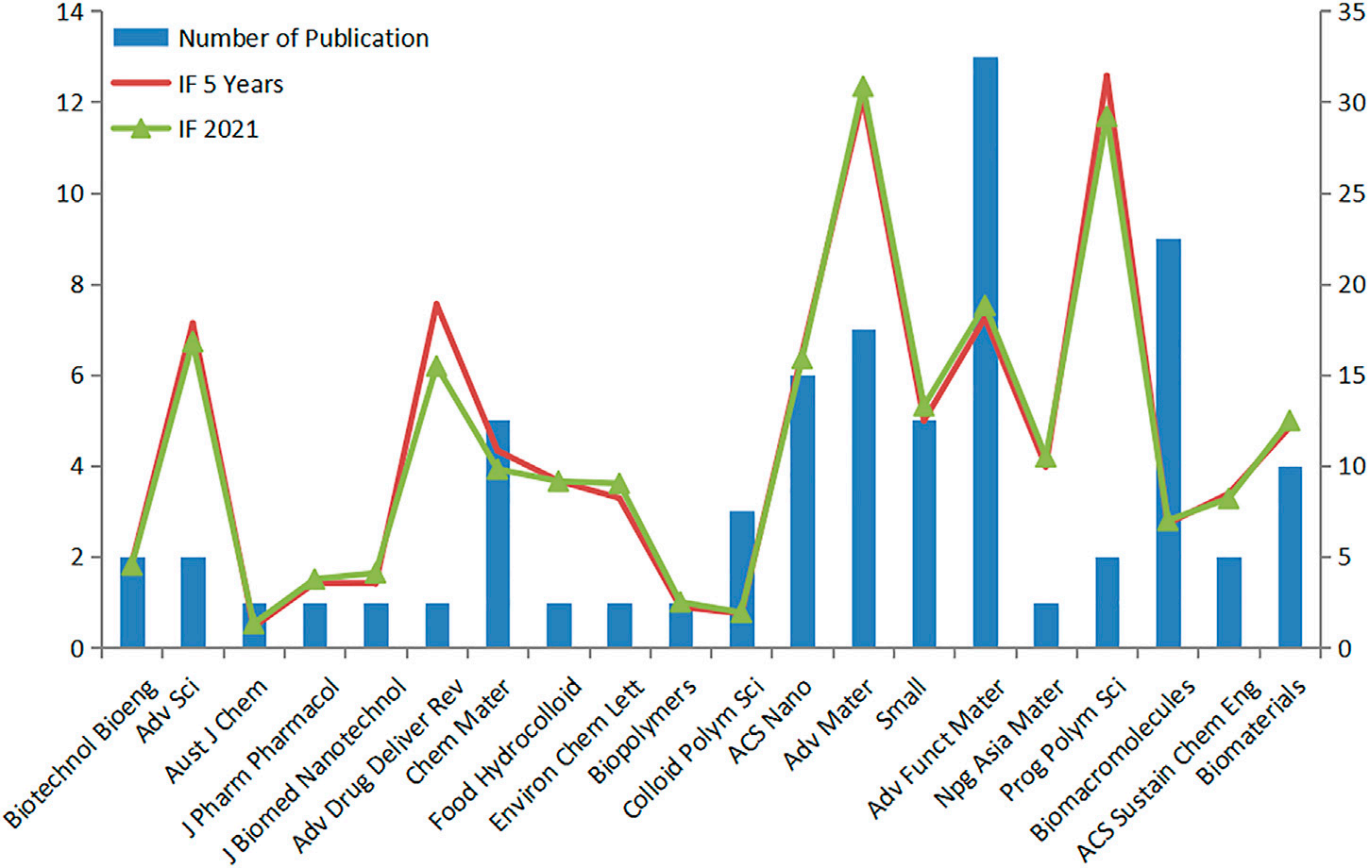
Top 20 journals ranked by the average citation rate.

**FIGURE 5:**
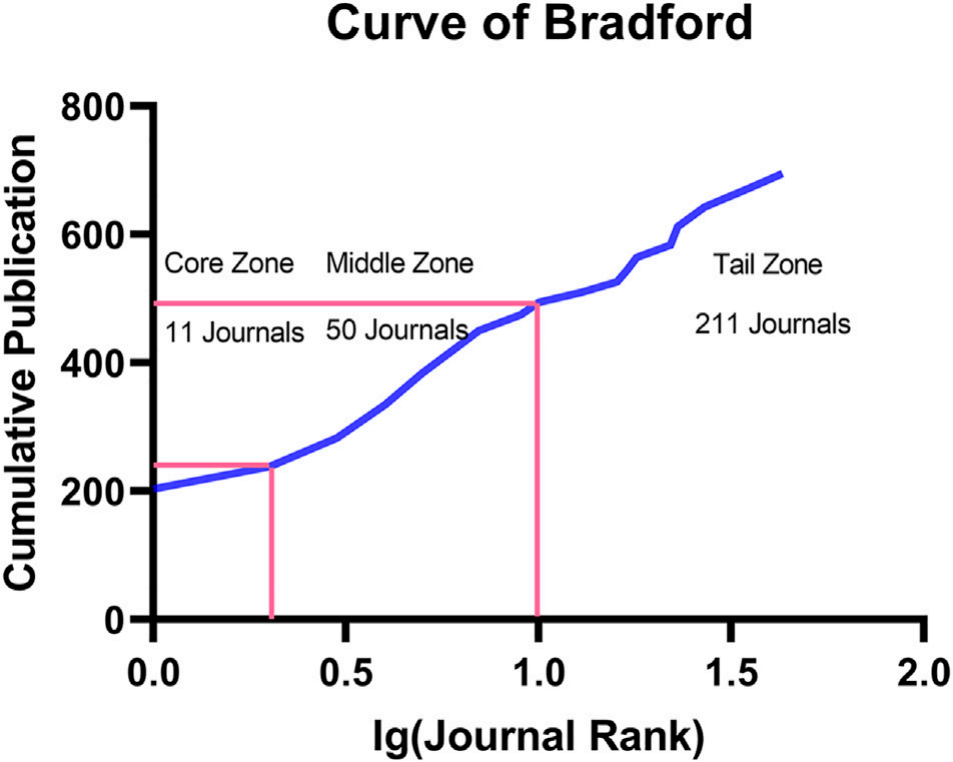
Bradford curve.

In Figure 4, according to the average citation rate, we rank the published journals in this field in descending order, and a total of 20 journals are included. This picture summarizes the journals with high academic levels in this field, and we can intuitively obtain the number of published journals in this field from 2010 to 2022 and the impact factors of these journals. Among the 20 journals, the journal *Advanced Materials* had the highest impact factor (IF = 30.849), and the journal *Advanced Functional Materials* had the highest number of publications in this field (publication = 13). Then, we counted the number of publications in each journal and drew the Bradford dispersion curve. The results are shown in Figure 5. According to Bradford’s curve, we obtained 11 core-zone journals, 50 middle-zone journals, and 211 tail-zone journals. The core-zone journals included *Int J Biol Macro*, *ACS Appl Material Inter*, and *Carbohyd Polym*.

### Analysis of Authors and Cited Authors

**FIGURE 6:**
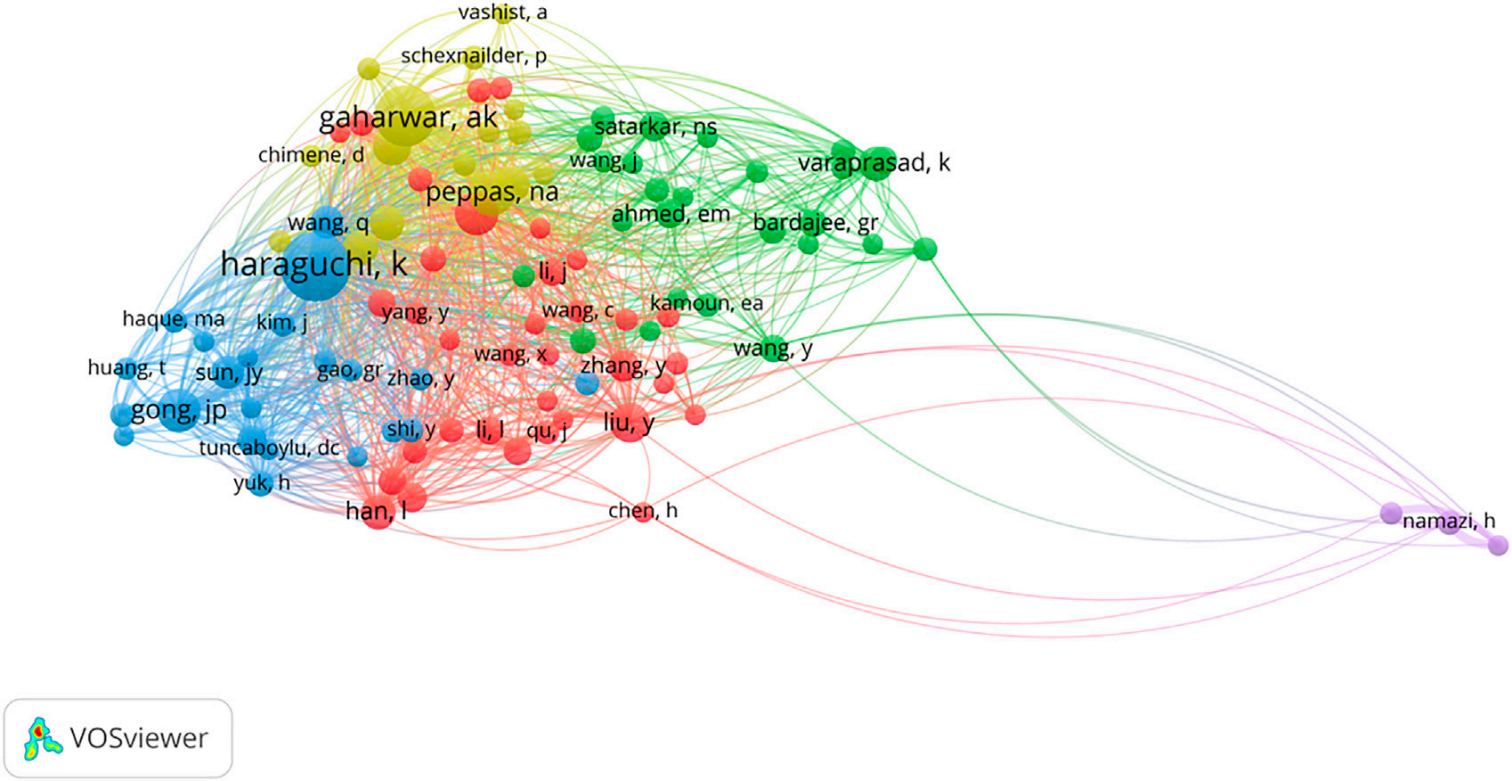
VOSviewer network visualization map based on co-cited authors in nanocomposite hydrogel.

Concerning authors, the top 10 productive authors and co-cited authors are listed in Table 1. From the 714 selected publications, Varaprasad K (11 articles) from Chile was the most prolific author among all 3,186 authors. One of his articles reported that inorganic biodegradable NHs can be used effectively as antimicrobial agents in the biomedical field by adding silver nanoparticles (Jayaramudu et al., 2013). Figure 6 visualizes the author’s co-cited network generated by VOSviewer. The figure shows that Haraguchi K (339 citations) from Mat Chem Lab was the most frequently co-cited author, and Gaharwar AK (257 citations) actively participated in research in this field, securing second place in both the top 10 productive authors and co-cited authors.

**TABLE 1:** Top 10 productive authors and co-cited authors.

Rank	Author	Count	Rank	Co-cited author	Citation
1	Varaprasad K	11	1	Haraguchi K	339
2	Gaharwar AK	10	2	Gaharwar AK	257
3	Liu Y	9	3	Peppas NA	138
4	Sadiku R	8	4	Gong JP	136
5	Namazi H	7	5	Yang J	130
6	Wang Y	7	6	Liu Y	113
7	Yang J	7	7	Shin SR	103
8	Bardajee GR	6	8	Lee KY	88
9	Hoare T	6	9	Hoffman AS	87
10	Rao KSVK	6	10	Varaprasad K	87

### Co-citation Analysis Based on Journals and References

**FIGURE 7:**
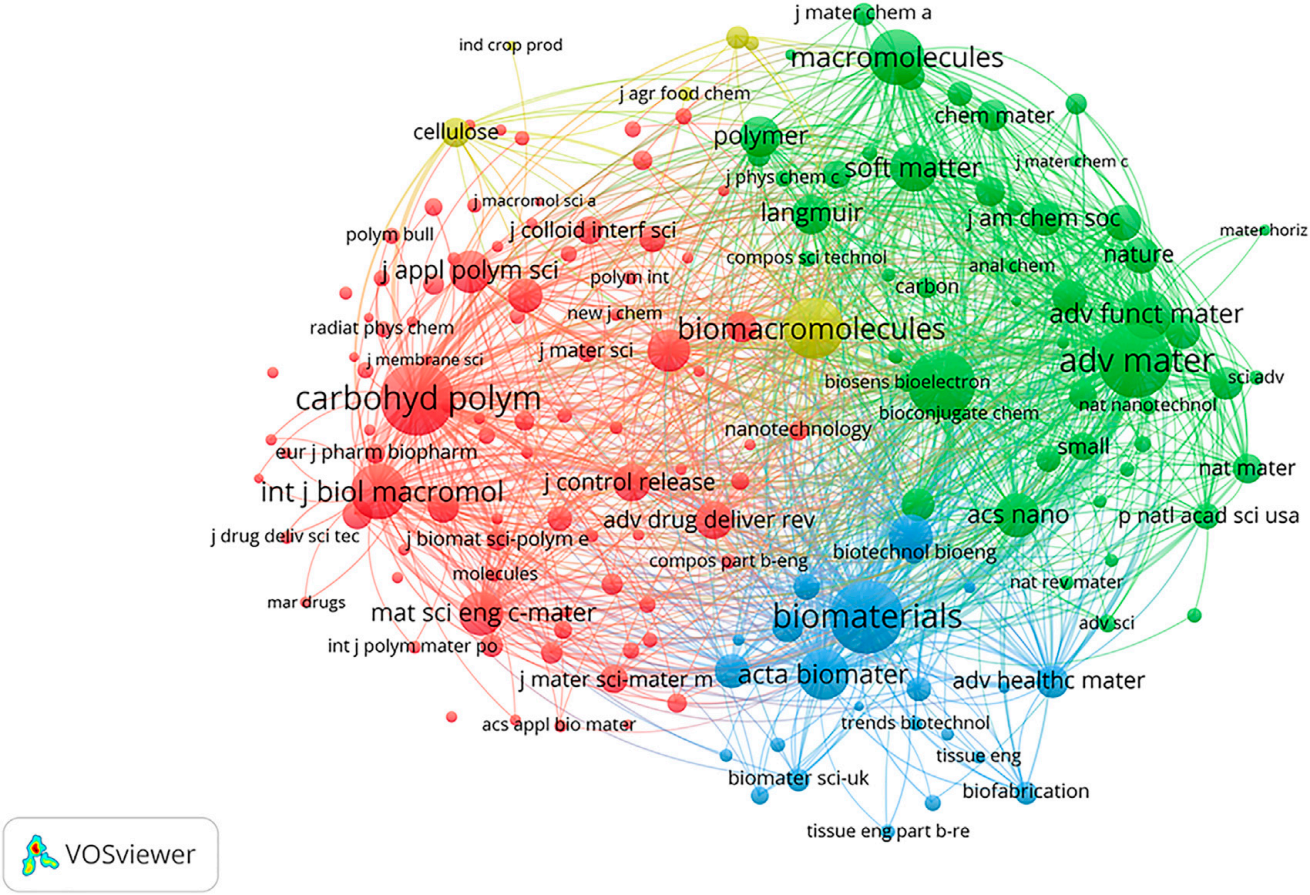
Co-citation network of journal references about nanocomposite hydrogel and related research. Co-citation analysis of journals.

The co-citations in journals and references are shown in Figure 7. The larger nodes give the cue that the magazine or author is cited more frequently. The thickness of the line represents the co-citation frequency, and in Figure 7, different colors represent different journal types. Therefore, journals such as *Carbohyd Polym*, *Adv Mater*, and *Biomaterials* are cited more frequently. Co-citation analysis of journals can tell readers which journals have made outstanding contributions in this field. Table 2 shows the top 10 co-cited references in the biomedical application of NHs, their respective authors, and their cited frequency.

**TABLE 2:** Top 10 co-cited references in the biomedical application of NH.

Rank	Cited frequency	Author	Year	Title
1	642	Gaharwar, AK	2014	Nanocomposite Hydrogels for Biomedical Applications Gaharwar et al. (2014)
2	641	De France, KJ	2017	Review of Hydrogels and Aerogels Containing Nanocellulose De France et al. (2017)
3	419	Han, L	2017	Mussel-Inspired Adhesive and Tough Hydrogel Based on Nanoclay Confined Dopamine Polymerization Han et al. (2017a)
4	360	Han, L	2017	A Mussel-Inspired Conductive, Self-Adhesive, and Self-Healable Tough Hydrogel as Cell Stimulators and Implantable Bioelectronics Han et al. (2017b)
5	325	Li, SQ	2018	Antibacterial Hydrogels Li et al. (2018)
6	290	Du, HS	2019	Cellulose nanocrystals and cellulose nanofibrils based hydrogels for biomedical applications Du et al. (2019)
7	249	Zheng, WJ	2015	Tough Al-alginate/Poly (N-isopropyl acrylamide) Hydrogel with Tunable LCST for Soft Robotics Zheng et al. (2015)
8	245	Peak, CW	2013	A review on tough and sticky hydrogels Peak et al. (2013)
9	242	Khalil, H	2016	A review on chitosan-cellulose blends and nanocellulose reinforced chitosan biocomposites: Properties and their applications Saurabh et al. (2016)
10	229	Zhu, CH	2012	Photothermally Sensitive Poly (N-isopropyl acrylamide)/Graphene Oxide Nanocomposite Hydrogels as Remote light-controlled Liquid Microvalves Zhu et al. (2012)

### Keywords Co-Occurrence Analysis

**FIGURE 8:**
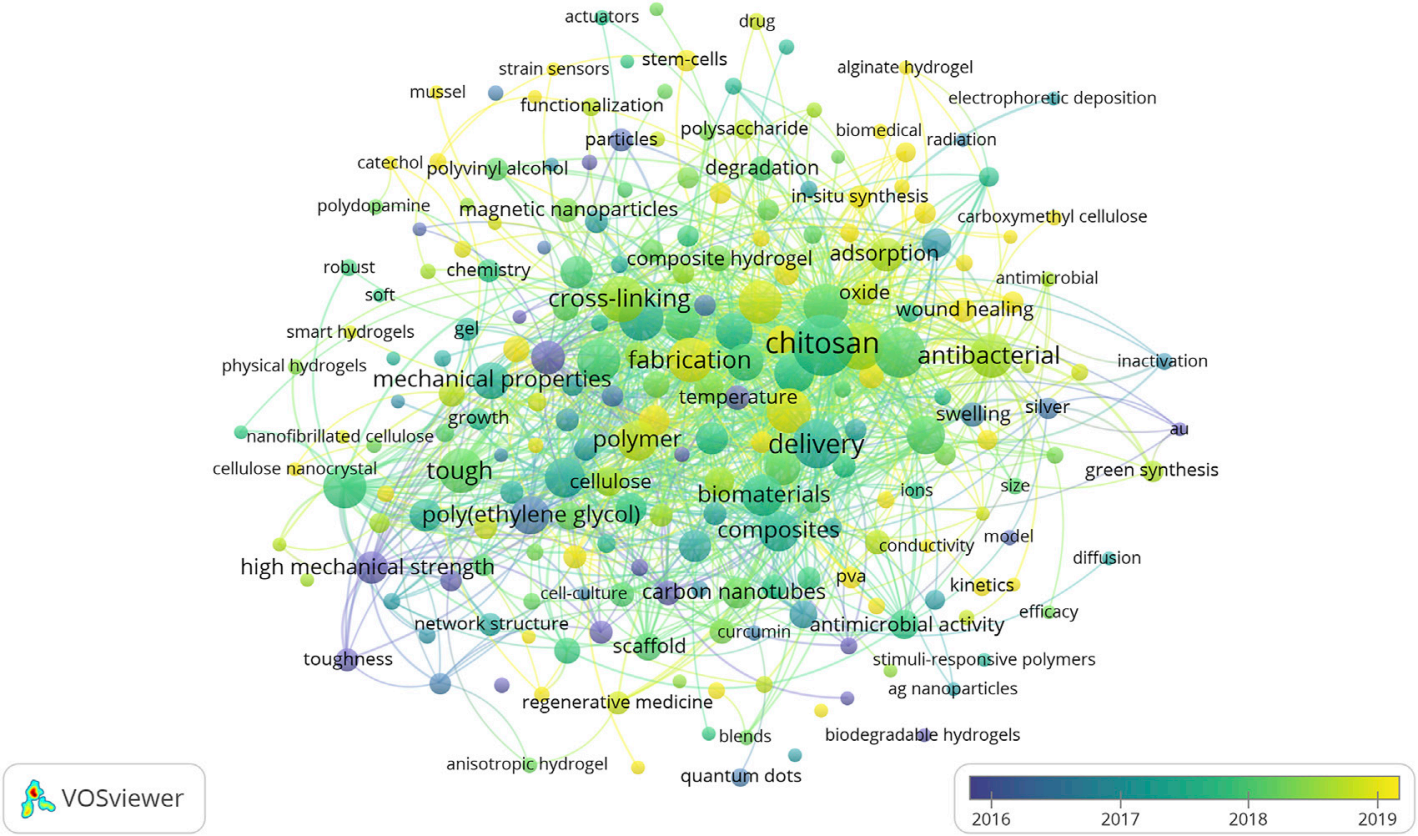
Visualization based on keyword co-occurrence relationship.

Keywords are single-morpheme words or phrases that can show the full meaning or core content of a piece of literature(Xiang et al., 2017). The visualization below effectively shows the tendencies and hot spots in this field, which provides massive useful information for researchers. After setting the threshold to 5 and deleting invalid keywords such as “hydrogel” and “release”, we obtained Figure 8 in the VOSviewer software, and after removing invalid keywords, we clarified the hot research directions of NHs, for example, the application in antibiosis, drug delivery, and so on. According to Figure 8, the larger the node is, the higher the frequency of these keywords in 713 papers. For example, among the 713 papers, nanocomposite hydrogels and hydrogels are the words with higher frequency, and the yellow node indicates the new keywords in recent years, which are helpful to predict the research hot spots and research directions in this field. For example, recent research hot spots include the application of 3D printing in this field and research on injectable hydrogels (Gorantla et al., 2019; Polat et al., 2020; Motealleh et al., 2021b).

## Discussion

In our current study, we analyzed 714 publications about NHs in the biomedical field indexed in the WOS core database to determine the recent research panorama in a bibliometric approach. Based on the trends from 1 January 2010 to 3 February 2022, we identified that the number of annual scientific publications about NHs showed an overall increasing trend, and the most productive period was 2021. The major kind of publication in the past 10 years was research articles (more than 77%), which showed that the applications of NHs were constantly explored and drew more attention in the biomedical field. According to the growth model that we fit, the number of papers increased linearly by year, which illustrated that NH research in the biomedical field is gradually maturing and coincided with upwards trends in the applications of nanometer materials. In this field, China, the United States, and India made outstanding contributions to the development of NH research. Among all the scientific institutions, Tabriz University of Medical Sciences, Chinese Academy of Sciences, and Tshwane University of Technology were active and contributed to the research front. Nonetheless, the interaction and cooperation around NHs in these leading countries and institutions were not compact, which revealed that there is great potential for collaborative research. Transnational and cross-team cooperation is the key to accelerating the balanced and comprehensive development of NHs around the world.

We concluded several main journals in the last 10 years based on the number of publications and average citation rate, including *Advanced Materials* with the highest impact factor (IF = 30.849), *Advanced Functional Materials* with the highest number of publications, and *Int J Biol Macro* with the most citations.

• Analysis of the co-citation map of authors showed that the articles of Haraguchi K, Gaharwar AK, Peppas Na, and a few other researchers impacted the trend and progress in NHs from 2010 to 2022. Among them, Haraguchi K is from Canada, Gaharwar Aki is from Japan, and Peppas Na is from America. These three countries are also mentioned in Figure 2 as countries with many publications about NHs. However, although China has the most publications in this field, its number of citations of the articles is less than that of the others. This may be because the start time of conducting research in this direction in China is later, and Chinese scholars are still enthusiastic about NH applications in biomedicine. The nanocomposite hydrogels for biomedical applications in biotechnology and bioengineering are the most cited references (Table 2). It concluded the most recent developments at that time in the field of nanocomposite hydrogels with emphasis on biomedical and pharmaceutical applications, published in 2014 (Gaharwar et al., 2014). In addition, Gaharwar AK and other researchers forecast that NHs in the future will tend to be improved in network performance by integrating suitable biological clues in addition to strictly controlling their physical properties. Moreover, the team of Dr. Gaharwar AK mentioned that researchers can tailor the properties of NHs for required applications and that this provides broad prospects for NH applications in the biomedical field (Gaharwar et al., 2014).The sixth category in Table 2 offers comprehensive literature on cellulose nanocrystal- and cellulose nanofibril-based hydrogels for biomedical applications, which provides a valuable outlook in this field and the deficiency of NHs in practical applications. In this review, the authors raised concerns about the biocompatibility and *in vivo* toxicity of cellulose nanocrystal (CNC)- and cellulose nanofibril (CNF)-based hydrogels, because most of the research on them is still in the preclinical stages and lacks relevant data. Therefore, more related clinical studies should be performed to fill the void in NH *in vivo* applications. It is also mentioned that emerging 3D-printing techniques may provide a possibility for the large-scale production of NHs.

Keyword co-occurrence analysis reflected the developing trends and hot spots of NHs (Figure 8). Due to the special construction and physical properties of NHs we mentioned above, the hot spots are *in vitro* drug delivery of NHs, injectable hydrogels, and the application of 3D printing, especially in the simulation of bone (Asadi et al., 2018; Gorantla et al., 2019; Du et al., 2021; Shahid et al., 2021; Syed Azhar et al., 2022). The keywords appearing in Figure 8 are connected closely and have strong centrality, which shows that the application of NHs has developed to different orientations continually and rapidly (Motealleh et al., 2021a).

## Limitations

Although we used a detailed retrieval strategy, there were certain limitations in the WOSCC. First, we chose the WOS database as a data source because it is considered to have standardized and comprehensive records compared with other databases (Polat et al., 2020). A large amount of literature is added to WOS every day, but only some of it is retrieved in WOSCC, causing a lack of data sources. Moreover, the language was limited to English, so this article does not include publications other than English publications. Additionally, as research continues to progress, our analysis is limited from January 1, 2010 to February 3, 2022, and future studies should add the latest articles to update the development. Because of limitations in our involved publications and the development in NH biomedical applications, there are fewer comprehensive studies in our study, and we are restricted to helping researchers. We hope that more in-depth analysis will be published in future studies to contribute to this field.

## Conclusion

There is no doubt that this study has provided useful and valuable information to summarize the progress of NH applications in the biomedical field. Visualized analysis via highly qualified research articles over the past 11 years helped us identify the current trends and hot spots in this field. An increasing number of studies have proven that the biomedical application of NHs is a potential and emerging trend, indicating that researchers should pay more attention to exploring the functions of NHs and providing new clinical treatment ideas.

## Data Availability

The raw data supporting the conclusion of this article will be made available by the authors, without undue reservation.
